# ENHANCING FEDERAL REVENUE FOR HOME AND COMMUNITY-BASED SERVICES UNDER THE AMERICAN RESCUE PLAN ACT

**DOI:** 10.1093/geroni/igac059.134

**Published:** 2022-12-20

**Authors:** Edward Miller, Lisa Beauregard

**Affiliations:** University of Massachusetts Boston, Boston, Massachusetts, United States; Massachusetts Executive Office of Elder Affairs, Boston, Massachusetts, United States

## Abstract

The American Rescue Plan Act (ARPA) includes a one year 10 percentage point increase in the Federal Medical Assistance Percentage for Medicaid-funded home and community-based services (HCBS). The goal is to strengthen state efforts to help older adults and people with disabilities live safely in their homes and communities rather than in institutional settings during the COVID-19 pandemic. This presentation provides a detailed description and analysis of this provision, including issues state governments need to consider when expending the additional federal revenue provided. It also draws lessons from the Affordable Care Act’s Balancing Incentive Program to suggest insights for the potential of ARPA to promote further growth in Medicaid HCBS programs. The presentation concludes by highlighting the importance of instituting strategies and processes for maximizing enhanced federal matching funds under ARPA in preparation for subsequent availability of substantial additional federal resources targeting Medicaid HCBS under other proposed initiatives.

